# A combined amplicon approach to nematode polyparasitism occurring in captive wild animals in southern China

**DOI:** 10.1186/s13071-024-06173-0

**Published:** 2024-02-28

**Authors:** Hongyi Li, Zhengjiu Ren, Weijian Wang, Fei Shen, Jingyi Huang, Chuyue Wang, Jinzhi Lu, Xi Pan, Lihua Xiao, Yaoyu Feng, Dongjuan Yuan

**Affiliations:** Guangdong Laboratory for Lingnan Modern Agriculture, College of Veterinary Medicine, South China Agricultural University, Guangzhou, 510642 China

**Keywords:** Captive wild animal, Gastrointestinal nematodes, Fecal examination, Amplicon

## Abstract

**Background:**

Gastrointestinal tract (GIT) nematodes prefer to live in the intestines of wild animals, causing damage and even death, and posing a zoonotic risk. The polyparasitism of GIT nematodes results in the complex dynamics of the nematode communities that occur naturally in wild animals. However, the nematode community in captive wild animals is poorly understood.

**Methods:**

We combined  microscopic examination and amplicon sequencing for community diversity.

**Results:**

We characterized GIT nematode assemblages to one order, one family, four genera, and ten species, in 512 fecal samples of 121 species from captive wild animals in southern China. The positive rate of GIT nematodes was 20.7% (106/512), including 42.3% (11/26) in reptiles, 26.5% (39/147) in herbivores, 25.0% (25/100) in non-human primates, 20.0% (5/25) in omnivores, 12.2% (9/74) in carnivores, and 12.1% (17/140) in avians. The dominant nematodes were *Haemonchus contortus* in herbivores and *Trichuris* species in primates. The nematode communities of arboreal primates differed from their terrestrial counterparts, reflecting both host phylogeny and ecological constraints. Soil-transmitted *Strongyloides* species were widespread throughout the herbivore, primate, avian, and carnivore communities, and tended to infect omnivorous primates and terrestrial herbivores. In addition, new *Trichuris* and *Heterakis* species were found in the nematode communities of captive porcupines and peafowls.

**Conclusion:**

This study highlights the variation in the composition of the GIT nematode community and strengthens the attention to the harms induced by zoonotic nematodes and co-infective nematodes with low species richness.

**Graphical Abstract:**

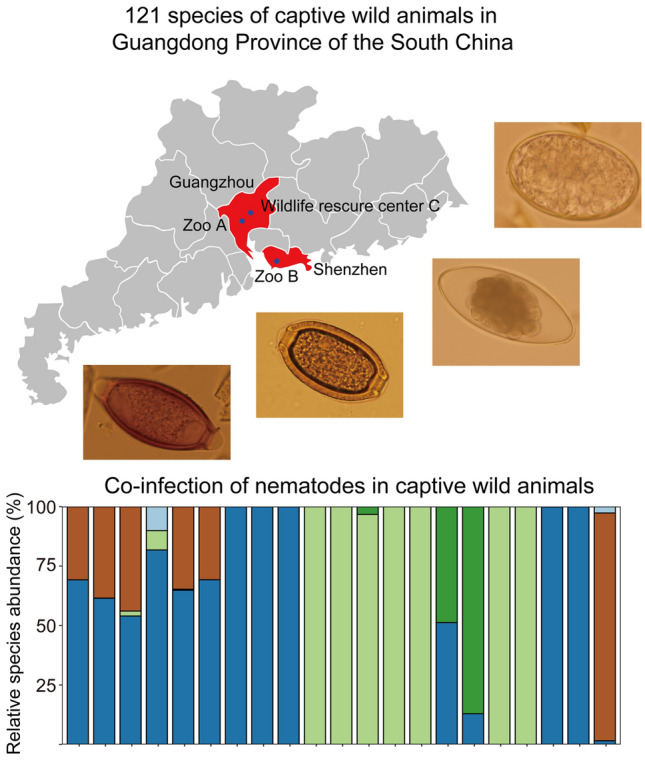

**Supplementary Information:**

The online version contains supplementary material available at 10.1186/s13071-024-06173-0.

## Background

Southern China is a subtropical region and hosts a great variety of wild animals, many of which live in nature reserves, zoos, or rescue centers that provide refuge for some of these endangered species. Most parasitic nematodes prefer to live in the gastrointestinal tract (GIT) of wild animals, including non-human primates, omnivores, herbivores, avians, reptiles, and carnivores [1]. Infection with these GIT nematodes causes diarrhea and emaciation or even death, affecting the population of wild animals and threatening species already on the brink of extinction. In addition, cross-transmission of some zoonotic species is detrimental to human health and a threat to public safety [2].

The major nematodes in wild animals can be grouped into clades I, II, III, IV, and V according to the small subunit ribosomal RNA (SSU rRNA) sequence [3]. The common GIT nematodes parasitized in wild animals include *Trichuris* and Capillariidae species from clade I, ascarid species in the Ascaridomorpha and filariae in Spiruromorpha from clade III, the facultatively parasitic *Strongyloides* species from clade IV, and the Strongylida species from clade V [4–6]. However, nematode polyparasitism, nematode life cycles, environmental conditions, host biological factors, and other aspects of host ecology result in the complex dynamics of the nematode community structure in wild animals [7]. The dominant species with high infection intensity can be easily identified, but the co-infective species remain unidentified or have been overlooked for some reasons. Therefore, this study on the nematode community investigates the polyparasitism of GIT nematodes in captive wild animals.

The eggs of GIT nematodes are found in the feces of their hosts, and microscopic examination of fecal eggs has been commonly used to identify nematode infection; however, polyparasitism of nematodes with similar egg shapes cannot be identified by microscopic examination, nor can it be detected by single-site identification. Amplicons of SSU rRNA containing variable sequence regions have been used to identify co-infective nematodes in the soil samples using next-generation sequencing (NGS) [8]. Illumina sequencing of internal transcribed spacer 2 (ITS2) amplicons has also been used to analyze the clade V nematodes in livestock and poultry [9]. However, amplicons commonly have a limited sequence length of ~350 base pairs (bp), which may be insufficient to effectively identify novel nematode species.

To fully identify GIT nematodes, we combined microscopic examination of fecal eggs, amplicon analysis, and polymerase chain reaction (PCR) identification to comprehensively survey GIT nematode communities in captive wild animals. To improve specificity and reduce fungal interference, the nematode-specific SSU rRNA motif was screened for amplicon analysis. Our goal was to approach the naturally occurring polyparasitism of GIT nematodes in captive wild animals present in southern China. The study provides clarity regarding the diversity of GIT nematode polyparasitism, which will motivate efforts to prevent and control GIT nematode diseases and protect wild animal and human health in southern China.

## Methods

### Ethics statement

All fecal samples used in this study were collected from captive wild animals with the permission of the zoo. All experimental procedures were in accordance with the Animal Ethical Procedures and Guidelines of the People’s Republic of China. The research protocol was reviewed and approved by the Ethics Committee of the South China Agricultural University, People’s Republic of China.

### Fecal sample collection and examination

Captive wild animals were housed in Zoo A in Guangzhou, Zoo B in Shenzhen, and the Wildlife Rescure Center (WRC) C in Guangdong. Zoo A and WRC C are 14 km apart, and Zoo A and Zoo B are 117 km apart. The two zoos and one rescue center included in this study were located in Guangdong Province. A total of 512 fecal samples from 121 wild animals were collected from these three sites between April 2019 and August 2022. The enclosures for the captive wild animals were cleaned every day. Each sample was collected from one animal and consisted of fresh feces collected from the ground. Each sample was collected individually and stored at 4 °C prior to microscopic examination, DNA extraction, and PCR analysis. Two Sprague Dawley (SD) rats aged 7 weeks were infected by subcutaneous injection with 3500 third-instar larvae (L3) of *Nippostrongylus brasiliensis* cultured in our laboratory. The feces of the infected rats were collected between 7 and 13 days post-infection (dpi). A sample of fresh rat feces was collected and divided equally. One half was used for DNA extraction and the other half was used to calculate eggs per gram of feces (EPG). Rat feces were soaked in saturated salt buffer and mixed well by vortexing. The number of floated eggs was calculated using a McMaster egg counting chamber. The morphological identification of the eggs was done by microscopic examination.

### DNA extraction and PCR amplification of SSU rRNA and ITS2 amplicons

Approximately 200 mg of feces from each positive sample was washed three times with distilled water by centrifugation at 2000×*g* for 10 min. Genomic DNA was extracted from the fecal samples of captive wild animals and SD rats utilizing the FastDNA^®^ SPIN Kit for Soil DNA (MP Biomedicals, Santa Ana, CA, USA), and then preserved at −20 °C. The SILVA database (https://www.arb-silva.de/) contains large subunit ribosomal RNA (23S/28S rRNA) and SSU rRNA sequences. It is frequently applied for rRNA amplicon analysis and nematode identification [10]. The silva_132_99_18S dataset downloaded from the SILVA database comprised 55,715 SSU rRNA sequences belonging to 7313 fungi and 2038 nematodes. The nematode-specific SSU rRNA motif was acquired by screening fungal and nematode SSU rRNA sequences using the MEME software (https://meme-suite.org/meme/tools/meme). Twelve barcodes 5 nucleotides (nt) in length were designed using DNA barcodes from the R package. The primers with barcodes were used to amplify ~350 bp of SSU rRNA and ITS2 amplicons from the total fecal DNA. The fragments were amplified from total nematode DNA using DreamTaq DNA Polymerase (Thermo Scientific, USA). The PCR reaction of SSU rRNA amplicons was 94 °C for 5 min denaturation; 45 cycles at 94 °C for 45 s, 52 °C for 30 s, and 68 °C for 30 s; followed by a final extension at 68 °C for 7 min. The PCR reaction of ITS2 amplicons was 95 °C for 3 min denaturation; 35 cycles at 95 °C for 30 s, 58 °C for 30 s, and 72 °C for 30 s; followed by a final extension at 72 °C for 10 min. Eighty-four SSU rRNA amplicons and 44 ITS2 amplicons were amplified from the total fecal DNA of captive wild animals from three sample sites. Fourteen samples of rat feces were used for quality control, in which microscopic examination and PCR identification revealed only the presence of *N. brasiliensis* eggs. Concentrations of SSU rRNA and ITS2 amplicons ranged from 385.6 to 482.6 ng/μl and 335.6 to 1154.7 ng/μl, respectively. PCR products from different samples were mixed at equal quantities and then subjected to Illumina sequencing by Personalbio (Shanghai, China). Additional file 1: Table S1 provides details on the samples.

### PCR amplification of *Trichuris* ITS1 and *Ascaridia*/*Heterakis* SSU rRNA

PCR primers for the *Trichuris* ITS1 gene were used to detect *Trichuris* species in the fecal samples [11]. The first round PCR reaction for the ITS1 gene of *Trichuris* species (~1080 bp) was performed at 94 °C for 1 min denaturation; 40 cycles at 94 °C for 1 min, 61 °C for 30 s, and 72 °C for 75 s; followed by a final extension at 72 °C for 10 min. The nested PCR reaction for the ITS1 gene of *Trichuris* species (~890 bp) was performed at 94 °C for 1 min denaturation; 35 cycles at 94 °C for 30 s, 55 °C for 30 s, and 72 °C for 75 s; followed by a final extension at 72 °C for 10 min. PCR primers for SSU rRNA (~724 bp) were used to identify *Ascaridia* and *Heterakis* species in the avian fecal samples [12]. The sequences of the PCR primers are shown in Additional file 1: Table S2. The nested PCR products of the ITS1 from *Trichuris* species and SSU rRNA from *Ascaridia*/*Heterakis* species were sequenced on an ABI 3730 Genetic Analyzer (Applied Biosystems, Foster City, CA, USA). Nucleotide sequences were assembled using ChromasPro v.1.32 (http://technelysium.com.au/ChromasPro.html) and aligned to reference sequences from GenBank for nematode identification using ClustalX v.2.1 (http://clustal.org).

### Bioinformatics analysis

Approximately 1.2 Gb and 1.8 Gb of raw data of SSU rRNA and ITS2 amplicons, respectively, were obtained using Illumina sequencing. Fastp was used to perform quality control on the raw data, resulting in 1.1 Gb and 1.7 Gb of high-quality data of SSU rRNA and ITS2 amplicons, respectively. The sequencing depth of the samples was up to approximately 26,000 × ~ 40,000×. Fastq-multx was used to split the data based on barcode sequences. The split data underwent assembly, noise reduction, redundancy reduction, and chimerism reduction using DADA2 of Qiime2 software (v.2022.2). Then, the feature sequences with 100% coverage and 99% identity were obtained by clustering analysis using vsearch of Qiime2 software (v.2022.2). All feature sequences were subjected to blast analysis using the nucleotide Basic Local Alignment Search Tool (blastn) (v.2.12.0), and non-nematode sequences were removed using the parameter of –max_hsps 1 –evalue 1e−5. To remove the low-frequency nematode sequences, the R package ggplot2 was used to plot the relative species richness based on the frequency of *N. brasiliensis* feature sequences in the total frequency of the samples. The standard curves of SSU rRNA (*y* = 124.42*x*^−0.902^, *R*^2^ = 0.8641) and ITS2 (*y* = 1301.6*x*^−1.073^, *R*^2^ = 0.9606) of *N. brasiliensis* in rat feces were constructed to calculate the threshold of the captive wild animal samples (Additional file 1: Fig. S1).

### Statistical analysis

To understand the differences in the positive rate of GIT parasitic nematodes in the fecal samples of different captive wild animals, SPSS software was used for multivariate statistical analysis of the samples. The *t*-test and one-way analysis of variance (ANOVA) were used to evaluate differences in the positive rate of the captive wild animals. Spearman’s correlation coefficient and partial correlation coefficient were used. Analyses were performed using SPSS 16.0, and *P*-values < 0.05 were considered statistically significant.

## Results

### Captive wild animals in southern China

**Fig. 1:**
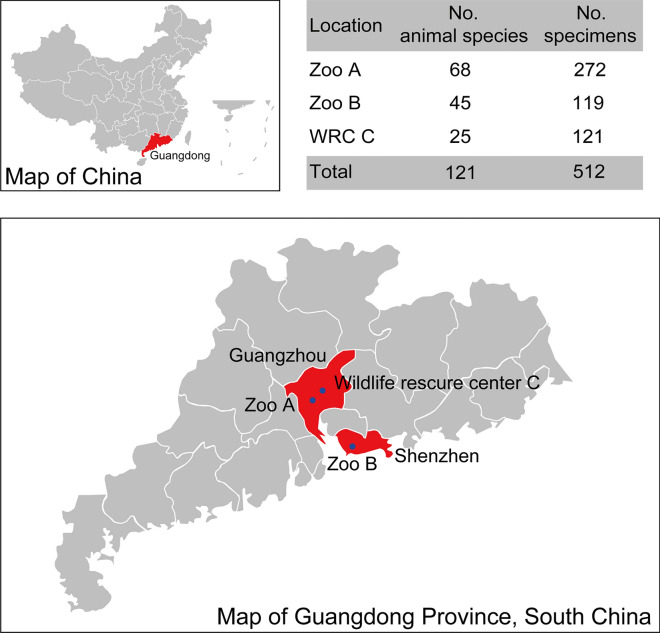
Map of Guangdong Province in southern China. Red regions are the map of Guangzhou and Shenzhen cities. Dark blue cycles are the sample sites

A total of 512 samples were collected from two zoos of Guangzhou and Shenzhen, and one wildlife rescure center of Guangzhou in Guangdong Province in southern China, as shown in Fig. 1. The enclosures of Zoo A were cleaned daily and anthelmintic treatment was performed twice in 1 year, and 272 fecal samples of captive wild animals were collected from Zoo A. Zoo B and WRC C were cleaned daily and anthelmintic treatment was not performed in 1 year, and 119 and 121 fecal samples were collected from Zoo B and WRC C, respectively. The species and sample numbers are shown in Additional file 1: Table S1.

### GIT nematode identification by microscopic examination, amplicon NGS, and PCR detection

The eggs in the animal feces from three sampling sites were identified by saturated saline floating and microscopic examination. The positive rate of parasitic nematode eggs was 19.9% (54/272) in 68 species of captive wild animals from Zoo A, 25.2% (30/119) in 45 species from Zoo B, and 18.2% (22/121) in 25 species from WRC C, thus, the average positive rate of nematodes was 20.7% (106/512) in total captive wild animals (Table 1), which was similar in these three sampling sites.

**Table 1:** Detection of nematodes in the feces of 121 species of captive wild animals from three sites in Guangdong Province, South China

Animal type	No. animal species/no. of specimens			No. positive/no. specimens (%)
	Zoo A	Zoo B	WRC C	
Herbivore	19/89	9/48	2/10	26.5 (39/147)
Carnivore	12/52	11/16	1/6	12.2 (9/74)
Omnivore	7/25	–	–	20.0 (5/25)
Non-human primate	14/38	16/43	3/19	25.0 (25/100)
Reptile	4/18	–	4/8	42.3 (11/26)
Avian	12/50	9/12	15/78	12.1 (17/140)
Total	68/272	45/119	25/121	20.7 (106/512)

Sequencing of nematode SSU rRNA and ITS2 amplicons was conducted to analyze the nematode community in captive wild animals. The primers for SSU rRNA amplicon were designed based on nematode-specific motifs by screening the fungal and nematode SSU rRNA sequences in the SILVA database. The primers NC1 and NC2 for ITS2 amplicon were used to analyze the common Strongylida species in captive wild animals. Combined with egg shape and PCR detection, six representative sequences were primarily identified from the SSU rRNA and ITS2 amplicons, respectively. Six categories of representative sequences from the SSU rRNA amplicons included *Ascaridia* or *Heterakis* species from avians, *Wellcomia* species from porcupines, *Trichuris* species from non-human primates, Oxyuridomorpha species from reptiles, *Strongyloides* species and *Toxascaris leonina* from South China tigers. Six categories of representative sequences in the ITS2 amplicons included *Haemonchus contortus* and *Cooperia* species from herbivores, *Oesophagostomum aculeatum* from monkey, *Trichostrongylus colubriformis* from antelope*, Ancylostoma ceylanicum* from tiger and bear, *Nematodirus helvetianus* from impala (Additional file 1: Tables S3 and S4)*.* As for widely parasitized *Trichuris* and poultry important *Ascaridia* and *Heterakis*, PCR fragments of *Trichuris* ITS1 and avian *Ascaridia* and *Heterakis* SSU rRNAs were further used to identify nematode species.

### Nematodes in non-human primates

**Fig. 2:**
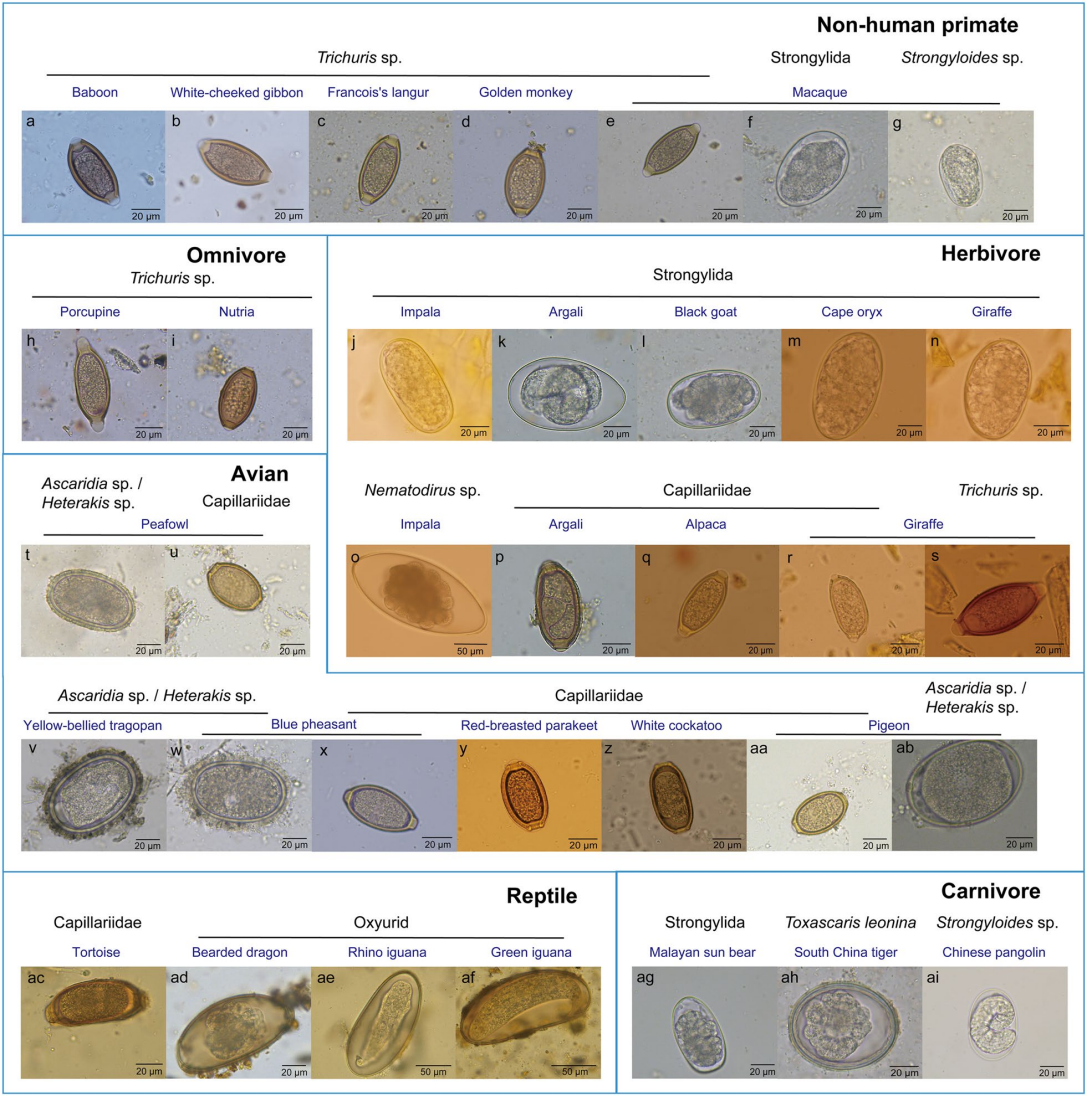
Microscopic examination of nematode eggs in the feces of captive wild animals. In non-human primates, *Trichuris* sp. (**a**–**e**) from baboon, white-cheeked gibbon, Francois’s langur, golden monkey, macaque; Strongylida (**f**) and *Strongyloides* sp. (**g**) from macaque. In omnivores, *Trichuris* sp. (**h**, **i**) from porcupine and nutria. In herbivores, Strongylida (**j**–**n**) from impala*,* argali, black goat, cape oryx, giraffe; *Nematodirus* sp. (**o**) from impala*,* Capillariidae (**p**–**r**) from argali, alpaca, giraffe; and *Trichuris* sp. (**s**) from giraffe*.* In avians, *Ascaridia* sp./*Heterakis* sp. (**t**) and Capillariidae (**u**) from peafowl; *Ascaridia* sp./*Heterakis* sp. (**v**, **w**) from yellow-bellied tragopan and blue pheasant; Capillariidae (**x**–**aa**) from blue pheasant, red-breasted parakeet, white cockatoo, pigeon; *Ascaridia* sp./*Heterakis* sp. (**ab**) from pigeon*.* In reptiles, Capillariidae (**ac**) from tortoise, Oxyurid (**ad**–**af**) from bearded dragon, rhino iguana, and green iguana. In carnivores, Strongylida (**ag**) from the Malayan sun bear, *Toxascaris leonina* (**ah**) from the South China tiger, *Strongyloides* sp. (**ai**) from the Chinese pangolin

**Fig. 3:**
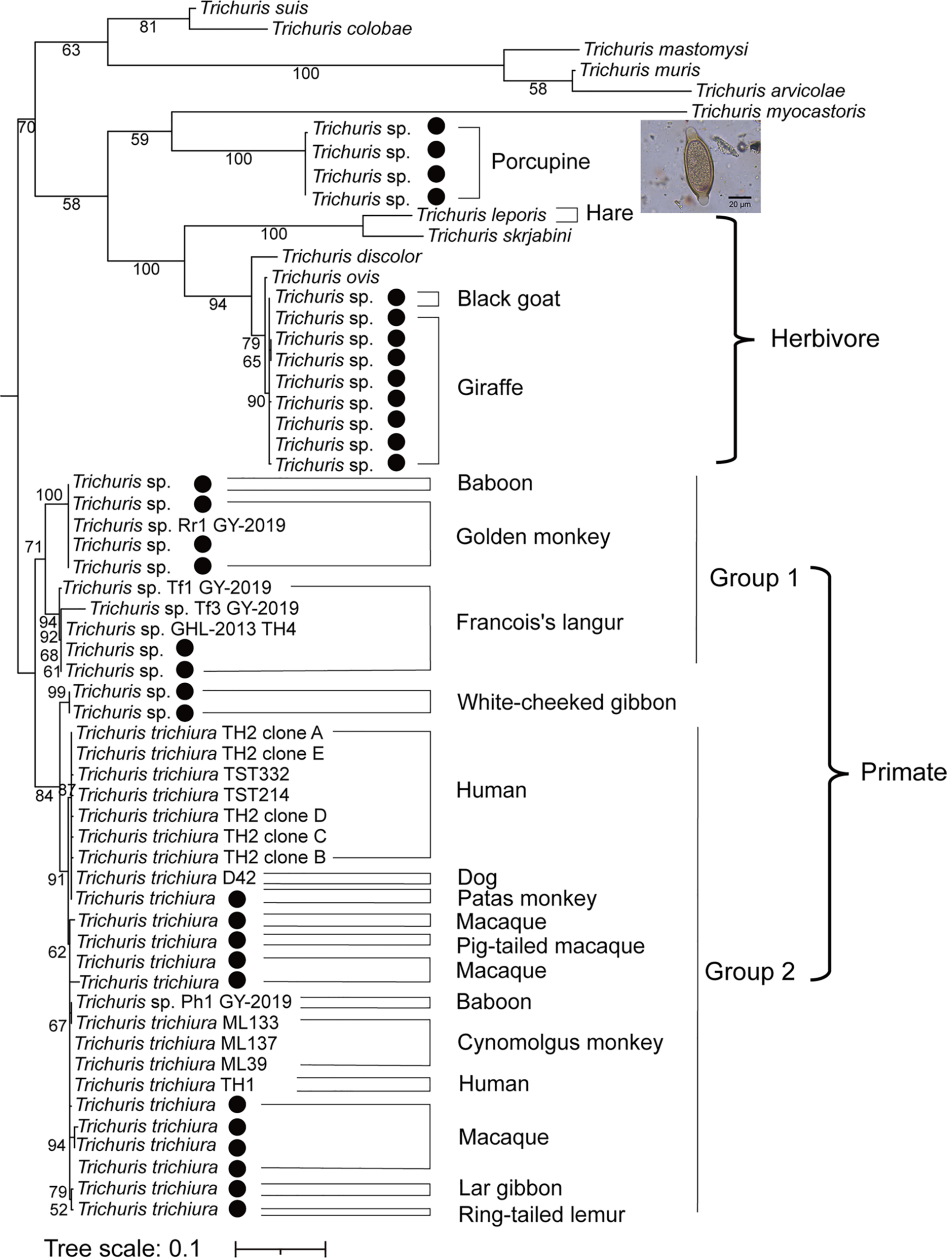
Phylogenetic relationship of *Trichuris* species from animals based on a maximum-likelihood analysis of ITS1 sequences. Bootstrap values were generated using 1000 replicate analyses. Nodes with bootstrap support values of 50 or greater were indicated. Representative sequences obtained from this study were indicated by black dots

Among 100 fecal samples from 26 primate species, the positive rate of nematodes by microscopic examination was 25.0% (25/100). The brown, barrel-shaped, and thick-shelled eggs with a pair of plugs at each end were found in the fecal samples of baboon, white-cheeked gibbon, Francois’s langur, golden monkey, and macaque. These eggs were 58.8 ± 7.7 μm long and 27.8 ± 2.7 μm wide and were identified as *Trichuris* species (Fig. 2a–e). The SSU rRNA or ITS2 amplicons were obtained by PCR amplification in 21 out of 25 positive samples. The results showed that *Trichuris* species had a detection rate of 57.1% (12/21) but could not be identified to the species level. Amplification of ~890 bp *Trichuris* ITS1 was performed to identify *Trichuris* species in 32 fecal samples from captive wild animals. Phylogenetic analysis showed that primate *Trichuris* were clustered together and divided into groups 1 and 2 (Fig. 3). *Trichuris* in group 1 were indeterminate species from non-human primates, and *Trichuris* from the same hosts were clustered in one branch. In group 2, *Trichuris* ITS1 from white-cheeked gibbon shared 92.7% sequence identity with human *Trichuris*, and *Trichuris* ITS1 from macaque, patas monkey, lar gibbon, cynomolgus monkey, and baboon shared more than 98.4% sequence identity with human *Trichuris* and were more closely clustered with human *Trichuris.* Thus, *Trichuris* species in non-human primates could be classified into two groups, some of which had the potential of zoonotic risk.

The Strongylida or *Strongyloides* eggs are oval-shaped and thin-shelled, and the size of the Strongylida egg is larger than that of the *Strongyloides* species, thus, the eggs in the macaque feces were identified as Strongylida (Fig. 2f) and *Strongyloides* species (Fig. 2g), respectively. As for Strongylida species, egg shape and amplicon analysis showed that *O. aculeatum* was detected in the feces of macaques and stump-tailed monkeys with a positive rate of 28.6% (6/21); and *H. contortus* in the feces of one macaque and one stump-tailed monkey had the relative species richness of 10.1% and 2.6%, respectively. The positive rate for *Strongyloides* was 66.7% (14/21) in the primate fecal samples, presumably infected with *Strongyloides stercoralis* or *Strongyloides fuelleborni*. These two species had similarly shaped eggs and could not be differentiated. In addition, Oxyuridomorpha species of clade III were found by amplicon analysis in the feces of golden monkeys and Francois’s langurs.

Non-human primates, especially the omnivorous macaque, were mainly co-infected with *Trichuris* species, *Strongyloides* species*,* or *O. aculeatum* (Table 2). Among 11 co-infected samples, three nematode species coexisted in the five fecal samples of macaque and stump-tailed monkey; *Strongyloides* and *O. aculeatum* coexisted in the six fecal samples of macaque*.* The results showed that *Trichuris* species had the highest positive rate of 23.0% in non-human primates, associated with 11% of human *Trichuris trichiura*; and *Strongyloides* and *O. aculeatum* had the positive rates of 14.0% and 6.0% in non-human primates, respectively (Table 2). In golden monkeys, white-cheeked gibbons, baboons, and patas monkeys, coexisting *Trichuris* species had the highest species richness, but in macaques, ring-tailed lemurs, and cynomolgus monkeys, coexisting *Strongyloides* species had the highest species richness, and the relative species richness of coexisting *Strongyloides* species was 28.0% higher than that of *O. aculeatum* in macaques. Thus, non-human primates were predominantly infected with *Trichuris* and *Strongyloides* species, especially *Trichuris* species.

**Table 2:** Summary of microscopic examination, amplicon analysis, and PCR identification of nematodes in 106 captive wild animals

Animal types	Numbered with letter	Nematode species	Detection rate (%)	Captive wild animals (detected nematodes)	Co-infective samples
Herbivore	A	*Haemonchus contortus*	21.1 (31/147)	Impala (A, B, D, G), cape oryx (A, B, D, F), giraffe (A, B, C, E), black goat (A, B, C, F), suri alpaca (A, B), blue sheep (A, B, H), big-eared goat (A, B), argali (A, B, E)	26
	B	*Strongyloides*	7.5 (11/147)		
	C	*Trichuris ovis*	6.1 (9/147)		
	D	*Cooperia*	3.4 (5/147)		
	E	Capillariidae	3.4 (5/147)		
	F	*Trichostrongylus colubriformis*	2.0 (3/147)		
	G	*Nematodirus helvetianus*	0.7 (1/147)		
	H	Oxyuridomorpha	0.7 (1/147)		
Non-human primate	A	*Strongyloides*	14.0 (14/100)	Stump-tailed monkey (A, D, F), macaque (A, B, C, D, F), cynomolgus monkey (A, B), golden monkey, Francois's langur (A, B, E), baboon (B), pig-tailed macaque, patas monkey, white-cheeked gibbon, ring-tailed lemur (A, C)	14
	B	*Trichuris*	12.0 (12/100)		
	C	*Trichuris trichiura*	11.0 (11/100)		
	D	*Oesophagostomum aculeatum*	6.0 (6/100)		
	E	Oxyuridomorpha	3.0 (3/100)		
	F	*Haemonchus contortus*	2.0 (2/100)		
Avian	A	Capillariidae	5.7 (8/140)	Blue pheasant (A, B, D), peafowl (A, B), pigeon (A, C), red-breasted parakeet, white cockatoo, silver pheasant (A), yellow-bellied tragopan, emu (B/C)	5
	B	*Heterakis gallinarum*	4.3 (6/140)		
	C	*Ascaridia nymphii*	2.1 (2/140)		
	D	*Strongyloides*	0.7 (1/140)		
Carnivore	A	*Toxascaris leonina*	5.4 (4/74)	South China tiger (A, B), Bengal white tiger, Malayan sun bear (B), lesser panda, Chinese pangolin (C)	1
	B	*Ancylostoma ceylanicum*	4.1 (3/74)		
	C	*Strongyloides*	4.1 (3/74)		
Omnivore	A	*Trichuris*	20.0 (5/25)	Porcupine (A, B), nutria (A)	2
	B	*Wellcomia*	8.0 (2/25)		
Reptile	A	Oxyuridomorpha	38.5 (10/26)	Rhino iguana, bearded dragon, green iguana (A), tortoise (B)	0
	B	Capillariidae	3.8 (1/26)		

### Nematodes in herbivores

Among 147 fecal samples from 27 herbivore species, the positive rate for nematodes was 26.5% (39/147). The SSU rRNA or ITS2 amplicons were obtained by PCR amplification in 34 out of 39 positive samples. The eggs of Strongylida species were found in impala, argali, black goat, cape oryx, and giraffe (Fig. 2j–n). Amplicon analysis showed that *H. contortus* was the dominant nematode in herbivores with a detection rate of 91.2% (31/34). *Cooperia* sp. was found in the feces of impala, cape oryx, and giraffe with a positive rate of 14.7% (5/34). *Trichostrongylus colubriformis* was found in the feces of cape oryx and black goat with a positive rate of 9.0% (3/34). The eggs in the impala feces had several embryonic cells and were relatively large and tapered at the two ends, which were primarily identified as *Nematodirus* species (Fig. 2o). Amplicon analysis further identified that *N. helvetianus* from Strongylida in the impala feces.

Results showed that the positive rate of *Strongyloides* (32.3%, 11/34) was lower than *H. contortus* and ranked second in herbivores, but the species richness was low. The *Strongyloides* in antelopes was presumed to be *Strongyloides papillosus,* which is a common species in ruminants. The yellow, thick-shelled eggs, containing a pair of plugs at each end and 55.2 ± 2.6 μm long and 28.9 ± 4.1 μm wide, from argali, alpaca, and giraffe were identified as Capillariidae species (Fig. 2p–r). The eggs of *Trichuris* species were found in the feces of giraffe (Fig. 2s)*.* Oxyuridomorpha and *Trichuris* species were found in the feces of blue sheep and giraffe, respectively.

Nine out of 17 samples coexisted with *H. contortus* and *Strongyloides* species, and the species richness of *H. contortus* was 78.4% higher than that of *Strongyloides* species. Six giraffe fecal samples coexisted with *H. contortus* and *Trichuris* species, and the species richness of *H. contortus* was higher than that of *Trichuris* species. *Trichuris* species from black goat and giraffe were clustered with herbivore-derived *Trichuris ovis*, *Trichuris discolor*, and *Trichuris skrjabini*, and hare-derived *Trichuris leporis* (Fig. 3). In three impala fecal samples, *Cooperia* species coexisted with *H. contortus*, and the species richness of *Cooperia* species was 64.8% higher than that of *H. contortus*. Thus, co-infection of two or more nematodes (26/39) was common in the positive samples from herbivores, and *H. contortus* was the dominant species with a positive rate of 21.1% (31/147) in herbivores (Table 2).

### Nematodes in avians

**Fig. 4:**
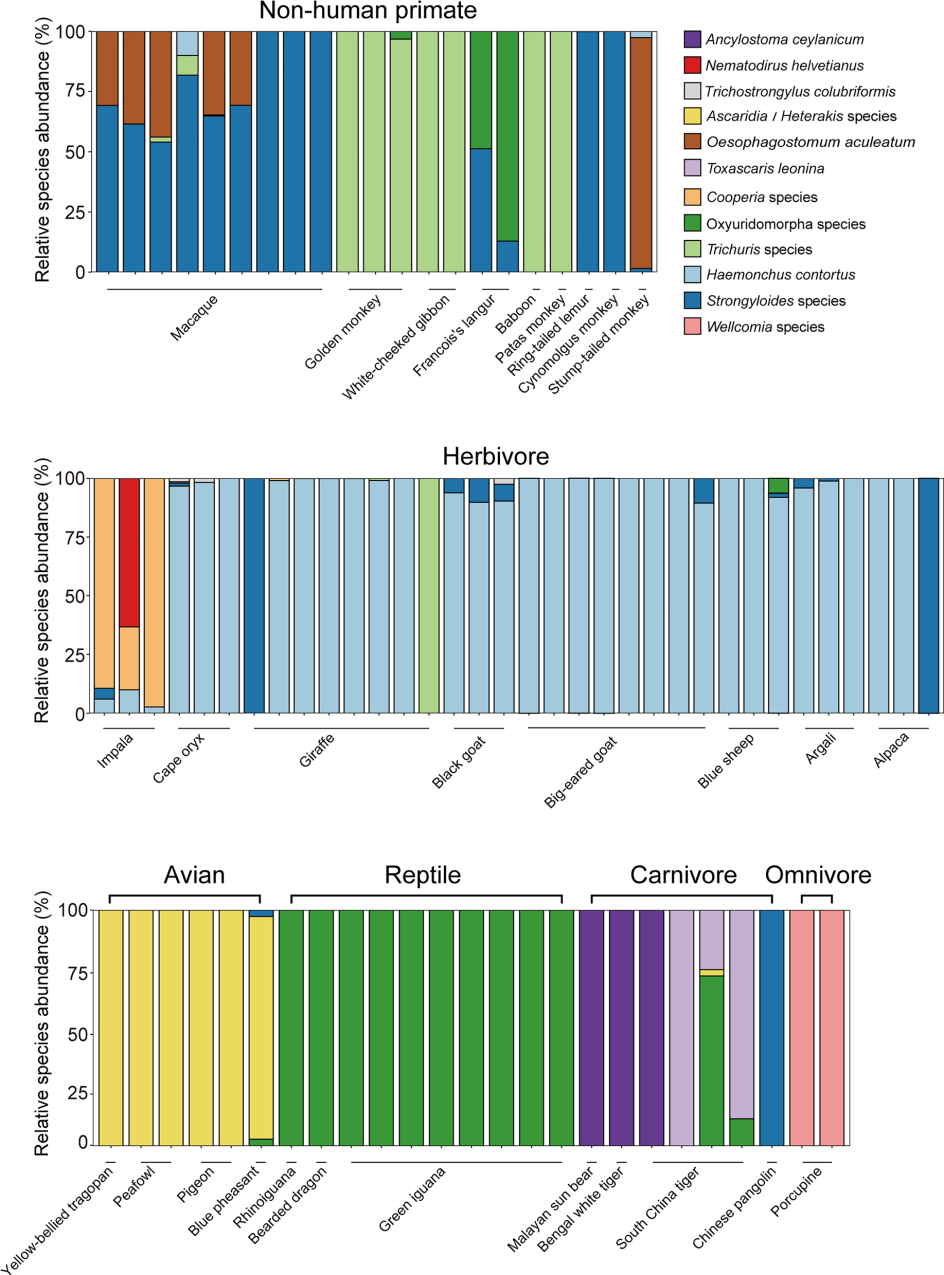
Relative species richness of SSU rRNA and ITS2 amplicons from captive wild animals. Representative sequences of SSU rRNA and ITS2 amplicons were used to analyze the relative species abundance of nematode species in captive wild animals

Among 140 fecal samples from 32 avian species, the positive rate for nematodes in avians was 12.1% (17/140). The *Ascaridia*/*Heterakis* eggs had a similar shape in the feces of peafowl (Fig. 2t), yellow-bellied tragopan and blue pheasant (Fig. 2v, w); and the Capillariidae eggs were found in peafowl (Fig. 2u), blue pheasant, red-breasted parakeet, white cockatoo, and pigeon (Fig. 2x–aa). SSU rRNA or ITS2 amplicons in six fecal samples could be identified among 17 positive samples. Amplicon analysis revealed three representative sequences in six avian fecal samples (Fig. 4). SSU rRNA amplicons could not clearly distinguish *Ascaridia* or *Heterakis* species. Thus, the results showed that peafowl, blue pheasant, and yellow-bellied tragopan of the family Phasianidae might be infected with *Ascaridia galli* or *Heterakis gallinarum*, while pigeons might be infected with *Ascaridia columbae* or *Ascaridia nymphii*. Blue pheasants may be co-infected with *Strongyloides avium*, *Ascaridia* species or *H. gallinarum,* and Capillariidae species.

**Fig. 5:**
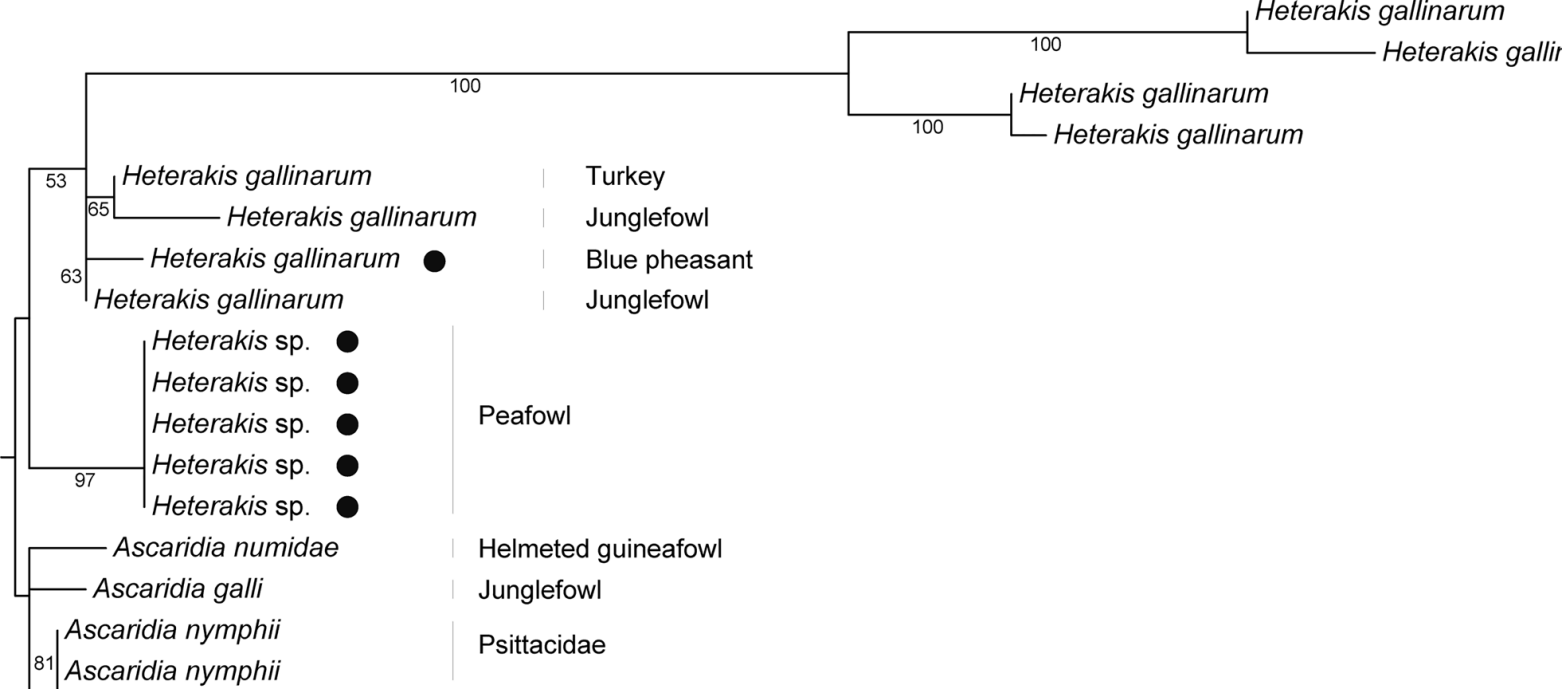
Phylogenetic relationship of avians *Ascaridia* in southern China based on maximum-likelihood analysis of SSU rRNA sequences using RAxML-ng software. Bootstrap values were generated using 1000 replicate analyses. Nodes with bootstrap support values of 50 or greater were indicated. Representative sequences obtained from this study were indicated by black dots

Approximately 730 bp of SSU rRNA fragments were further used to identify *Ascaridia* and *Heterakis* species [12]. The results showed that nematode SSU rRNA from blue pheasant was clustered with *H. gallinarum* from junglefowl, turkey, and pigeon in one branch (Fig. 5) and shared 100% sequence identity with *H. gallinarum* from jungle fowl, suggesting that the nematode in blue pheasant might be *H. gallinarum*. Nematode SSU rRNA obtained from peafowl was clustered on a branch with *H. gallinarum* and shared 98.0% sequence identity with known *H. gallinarum*, presumably identified as *H. gallinarum*. Nematode SSU rRNAs from pigeons were clustered in the avian Ascaridia branch and shared 100% sequence identity with *A. nymphii* from cockatiel, suggesting that pigeons may be infected with *A. nymphii*. The avians peafowl, blue pheasant, and pigeon were co-infected with *H. gallinarum*, *A. nymphii*, *Strongyloides*, and *Capillaria* species, and the positive rates of *Capillaria* species and *H. gallinarum* were 5.7% and 4.3%, respectively.

### Nematodes in other animals

In 26 fecal samples from eight reptile species, the positive rate for nematodes was 42.3% (11/26). The eggs of Capillariidae were found only in tortoise (Fig. 2ab), and the eggs of Oxyurid were observed in bearded dragons, rhino iguanas, and green iguanas (Fig. 2ac–ae). SSU rRNA or ITS2 amplicons from 10 fecal samples were identified by PCR amplification among 11 positive samples. Amplicon analysis showed that the Oxyuridomorpha species was found in the 10 fecal samples of bearded dragon, green iguana, and rhino iguana with a positive rate of 38.5% (10/26) (Table 2).

In 74 fecal samples from 21 carnivore species, the positive rate for nematodes was 12.2% (9/74). SSU rRNA or ITS2 amplicons from seven fecal samples were identified among nine positive samples. The Strongylida eggs were found in the Malayan sun bear (Fig. 2af). The oval, smooth, and thick-shelled eggs of *T. leonina* were found in the South China Tiger (Fig. 2ag). The thin-shelled eggs containing L1 larvae from the Chinese pangolin were identified as *Strongyloides* species (Fig. 2ah). Amplicon analysis showed that four representative sequences were found in seven carnivore fecal samples. *Ancylostoma ceylanicum* of the order Strongylida was detected in the fecal samples of the white tiger, South China tiger and Malayan sun bear. *Toxascaris leonina* was found only in the feces of the South China tiger with a positive rate of 5.4%. *Ascaridia*/*Heterakis* species, avian parasitic nematodes, in the feces of South China tigers may be due to the live chicken as food for South China tigers. In addition, *Strongyloides* species have been detected in the feces of the captive Chinese pangolin.

As for 25 fecal samples from seven omnivore species, *Wellcomia* species in the infraorder Oxyuridomorpha was found in porcupine, which was consistent with the previous study [13]. Oxyuridomorpha species was found in the feces of blue pheasant, South China tiger, reptile and porcupine, which may be derived from the ingested insects due to their feeding habits. *Trichuris* species was the dominant nematode in the observed omnivorous species with a positive rate of 20.0% (5/20) (Fig. 2h, i). The egg plugs of *Trichuris* species in porcupines were longer than those of other animals (Fig. 2h). Phylogenetic analysis showed that porcupine-derived *Trichuris* species were clustered in a branch that had the closest relationship to *Trichuris myocastoris* from nutria (Fig. 3). Thus, the porcupine-derived *Trichuris* species was presumed to be a novel species. *Trichuris* species in the observed omnivores, herbivores, and primates were diverse, but only a few species had been found and many novel species remain to be identified.

## Discussion

Southern China belongs to the subtropical climate with high precipitation, temperature, and humidity, creating conditions for the reproduction and growth of parasitic nematodes, especially for soil-transmitted nematodes. This region had abundant categories of captive wild animals that were widely parasitized by GIT parasitic nematodes [14, 15]. Some co-infective GIT nematodes with low species richness could also cause harm to their hosts, affecting wild animal biodiversity, and leading to zoonotic risk. Microscopic examination is commonly used to study nematodes, but only a limited number of nematode species can be identified. Nemabiome deep amplicon sequencing using ITS2 sequences is a useful approach to studying the GIT nematode communities of farm animals [9, 16]. In this study, improved methods of microscopic examination and deep sequencing of SSU rRNA and ITS2 amplicons were combined to elucidate the nematode community in 121 captive wild animals from 512 fecal samples in southern China.

### Identification of nematode community by integrated approaches

Fecal microscopy of eggs is a traditional and powerful method for rapid detection of GIT nematodes, in this study, it could not discriminate the eggs of Strongylida, *Trichuris*, Capillariidae, *Ascaridia,* and *Heterakis* species with similar shape in the fecal samples. In addition, ITS2 amplicons using primers NC1 and NC2 could identify Strongylida nematodes from clade V to the species level, but could not identify nematodes from other clades. A previous report showed that different regions of SSU rRNA amplicons used to detect soil nematodes covered 42.0% to 96.0% of nematode sequences and 45.3% to 96.6% of fungal sequences [17]. To improve specificity, SSU rRNA primers were designed on nematode-specific regions based on the fungal and nematode SSU rRNA sequences in the SILVA database. In this study, we mixed the same amount of DNA in each sample via PCR reactions and obtained 1.1 Gb and 1.7 Gb of SSU rRNA and ITS2 amplicon sequences using Fastp for quality control. The high sequencing depth of approximately 26,000 ×  ~ 40,000× would enlarge some nematodes with a very low level in the environment or in animal feces, even a very low level of nematodes originating from coprophagy or predator–prey (meal) in the animal feces. Similarly, non-nematodes and a very low level of nematodes originating from coprophagy or predator–prey would reach a high ratio in some fecal samples with a low level of EPG of infected nematodes. Thus, we used different EPG of *N. brasiliensis* in the ovulation curve of infected experimental rats to draw the standard curve. We calculated the frequency of *N. brasiliensis* and non-*N. brasiliensis* in the feces of experimental rats. The amounts of *N. brasiliensis* eggs were low during 7–8 dpi, and as a result, the frequency of non-*N. brasiliensis* was relatively high. The standard curve of *N. brasiliensis* in rat feces could reduce the effects of these very low levels of nematodes and improve the reliability of amplicon analyses. Furthermore, we combined microscopic examination of egg shape, PCR detection, and amplicon analyses to identify these infected nematodes in captive wild animals.

SSU rRNA amplicons were able to cover GIT nematodes of *Trichuris*, *Strongyloides*, *Heterakis*/*Ascaridia*, *Wellcomia*, Oxyuridae, and Strongylida to the genus or order level, but Capillariidae species could not be detected. PCR fragments of ~890 bp ITS1 and ~730 bp SSU rRNA were also used to identify *Trichuris* and *Ascaridia*/*Heterakis* species to the species level. The combination of microscopic examination, amplicon analysis, and PCR detection identified ten GIT parasitic nematodes to the species level, four to the genus level, one to the family level, and one to the order level.

### Flexibility for host selection of GIT nematodes in animal categories

The host range of most nematodes is not limited to a single host, suggesting variation in the dominant species in animal categories due to the possibility of host selection. *Trichuris* parasitized widely in omnivores (pigs and rodents), herbivores, and primates, but it had host selection of primates compared to other animals. Furthermore, *Trichuris* species was the dominant nematode in the arboreal primates of golden monkey and white-cheeked gibbon and the terrestrial primates of baboon and patas monkey, but terrestrial primates of macaque and cynomolgus monkey and arboreal primates of ring-tailed lemur had the dominant nematode of *Strongyloides* sp. Further analysis of *Trichuris* ITS1 PCR fragments showed that *Trichuris* spp. in primates were divided into two groups. *Trichuris* species in group 1 were found in golden monkeys and Francois's langurs, whereas *Trichuris* species in group 2 were found in humans, dogs, and some terrestrial and omnivorous non-human primates. As for *Trichuris* in monkeys, we could not exclude the possibility of environmental *Trichuris* species increasing the detection rate of *Trichuris* in monkeys. Although the two zoos and one rescue center were cleaned daily, the possibility remained for parasites in animal feces via the environment, coprophagy, or predator–prey transmission.

Soil-transmitted *Strongyloides* species were also widespread among primates, herbivores, avians, and carnivores. *Strongyloides* species had the highest species richness in some primates and also had relatively high species richness in giraffe, alpaca, and Chinese pangolin. Thus, *Strongyloides* species were more likely to infect omnivorous primates, some terrestrial herbivores, and burrowing pangolins.

*Haemonchus contortus,* a blood-sucking nematode, was the dominant species in antelope and giraffe among the observed herbivore species, but impala had higher species richness of *Cooperia* species than *H. contortus. A. ceylanicum* was the dominant species in the Malayan sun bear, Bengal white tiger, and South China tiger, but *T. leonina* and Oxyuridomorpha species were the dominant species in other South China tigers. Oxyuridomorpha species were also widely detected in the feces of stump-tailed monkey, blue pheasant, iguana, and porcupine, which used insects (the intermediate host of Oxyuridomorpha species) as food. Avians have always been used as food by the South China tiger. This feeding habit makes these wild animals susceptible to infection by Oxyuridomorpha species. Thus, the dominant species in the nematode community diverged in non-human primates and carnivores compared to herbivores, avians, and reptiles. The host selection of nematodes in primates and carnivores is a variation that could be influenced by the habitats and feeding habits of the host.

### Polyparasitism of GIT nematodes in animals

Polyparasitism is common in captive wild animals, but comprehensive studies of nematode communities are inadequate. In this study, *O. aculeatum*, *H. contortus*, and Oxyuridomorpha species might be co-infective nematodes with low species richness in primates. *Nematodirus helvetianus*, *Strongyloides*, *Cooperia*, and Capillariidae species were present in antelope and giraffe feces along with low species richness. Except for *H. gallinarum* and *A. nymphii*, co-infection with Capillariidae species in blue pheasants, peafowls, and pigeons should be of concern. These co-infective nematodes with low species richness still need further identification because some eggs might have originated from the environment, coprophagy, or predator–prey transmission. Furthermore, nematode communities in animal categories had limited stability, host phylogeny and ecology might be constraints in the composition and species richness of co-infective nematodes with low species richness in the nematode communities. In addition, co-infective nematodes with low species richness could aggravate harm to their hosts; similarly, the captive environment had the potential risk to cause these nematodes to infect other susceptible host animals during animal husbandry. Thus, although further study should be conducted to analyze these co-infective nematodes, the harms caused by co-infective GIT nematodes with low species richness must be of concern.

### New species for nematode biodiversity in animals

Wild animal is generally considered to be an important reservoir of pathogens. In this study, *Trichuris* species in porcupine feces had more prominent egg plugs than other known *Trichuris* eggs. PCR fragments of *Trichuris* ITS1 with ~890 bp were different from those available in the database, indicating that this may be a novel species. The ~724 bp SSU rRNA sequence of *H. gallinarum* from peafowl feces was different from that of *H. gallinarum* from blue pheasant, suggesting a novel strain of this species. Although the discovery of these new species in nematode communities from captive wild animals enriches the parasite biodiversity inventory, however, nematode biodiversity in wild animals faces host-driven co-extinction and redistribution through a combination of direct and indirect pressures in a changing climate [18].

### More concern on zoonotic nematodes in animals

Infection with zoonotic GIT nematodes causes symptoms of abdominal pain, diarrhea, anemia, and even death in humans. In this study, some species with zoonotic risk were found in a nematode community of captive wild animals. *Trichuris trichiura* from patas monkeys, macaques, pig-tailed macaques, ring-tailed lemurs, and lar gibbons were clustered with human *T. trichiura*, suggesting a possible zoonotic risk of this species. The presumed *Strongyloides* infecting non-human primates were either *S. stercoralis* or *S. fuelleborni*, both of which have a zoonotic risk. Zoonotic *T. colubriformis* has been found in antelopes. *Trichostrongylus colubriformis* infection causes severe diarrhea in the sheepherder [19]. Human infection with *T. colubriformis* has been reported in Laos, Thailand, Australia, and the United States [20]. Zoonotic* A. ceylanicum* has been found in fecal samples from South China tigers, white tigers, and Malayan sun bears, consistent with previous reports on brown bears and other felines in China [21]. The close relationship between humans and captive wild animals increased the zoonotic risk of *Trichuris*, *Strongyloides*, *T. colubriformis*, and* A. ceylanicum* in captive wild animals.

## Conclusions

Comprehensive analyses of GIT nematodes in 121 species of captive wild animals reveal that polyparasitism is common and the composition of the community structure varies in southern China. Host habitat and feeding habits may be one of the reasons for the host selection of nematodes in animals. Elucidating the nematode community in captive wild animals would strengthen the attention on the damage caused by co-infective GIT nematodes with low species richness.

### Supplementary Information

**Additional file 1: Figure S1.** Standard curve of egg shedding, SSU rRNA, and ITS2 effective sequence analyses. (A) Analysis of egg shedding of *N. brasiliensis* from SD rats. (B) SSU rRNA effective sequence analysis curve of *N. brasiliensis* from SD rats. (C) ITS2 effective sequence analysis curve of *N. brasiliensis* from SD rats. **Table S1.** Description of 512 fecal samples collected from 121 species of captive wild animals at three sampling sites in southern China. **Table S2.** PCR primers for all loci. **Table S3.** Six categories of representative sequences of SSU rRNA and ITS2. **Table S4.** Evaluation of the classification effect of the amplicon.

## Data Availability

Nucleotide sequence data reported in this paper are available in GenBank™ under the accession numbers OR502824–OR502855 (*Trichuris* species) and OR528248–OR528256 (*Ascaridia nymphii* and *Heterakis* species).
