# Supplementary material for: A combined amplicon approach to nematode polyparasitism occurring in captive wild animals in southern China
Source: Parasit Vectors. 2024 Feb 28;17:94. doi: 10.1186/s13071-024-06173-0 (PMC10900558; doi:10.1186/s13071-024-06173-0)
Supplement: Supplementary file 1 — Additional file 1: Figure S1. Standard curve of egg shedding, SSU rRNA, and ITS2 effective sequence analyses. (A) Analysis of egg shedding of N. brasiliensis from SD rats. (B) SSU rRNA effective sequence analysis curve of N. brasiliensis from SD rats. (C) ITS2 effective sequence analysis curve of N. brasiliensis from SD rats. Table S1. Description of 512 fecal samples collected from 121 species of captive wild animals at three sampling sites in southern China. Table S2. PCR primers for all loci. Table S3. Six categories of representative sequences of SSU rRNA and ITS2. Table S4. Evaluation of the classification effect of the amplicon. [file 13071_2024_6173_MOESM1_ESM.docx]

**Additional data**


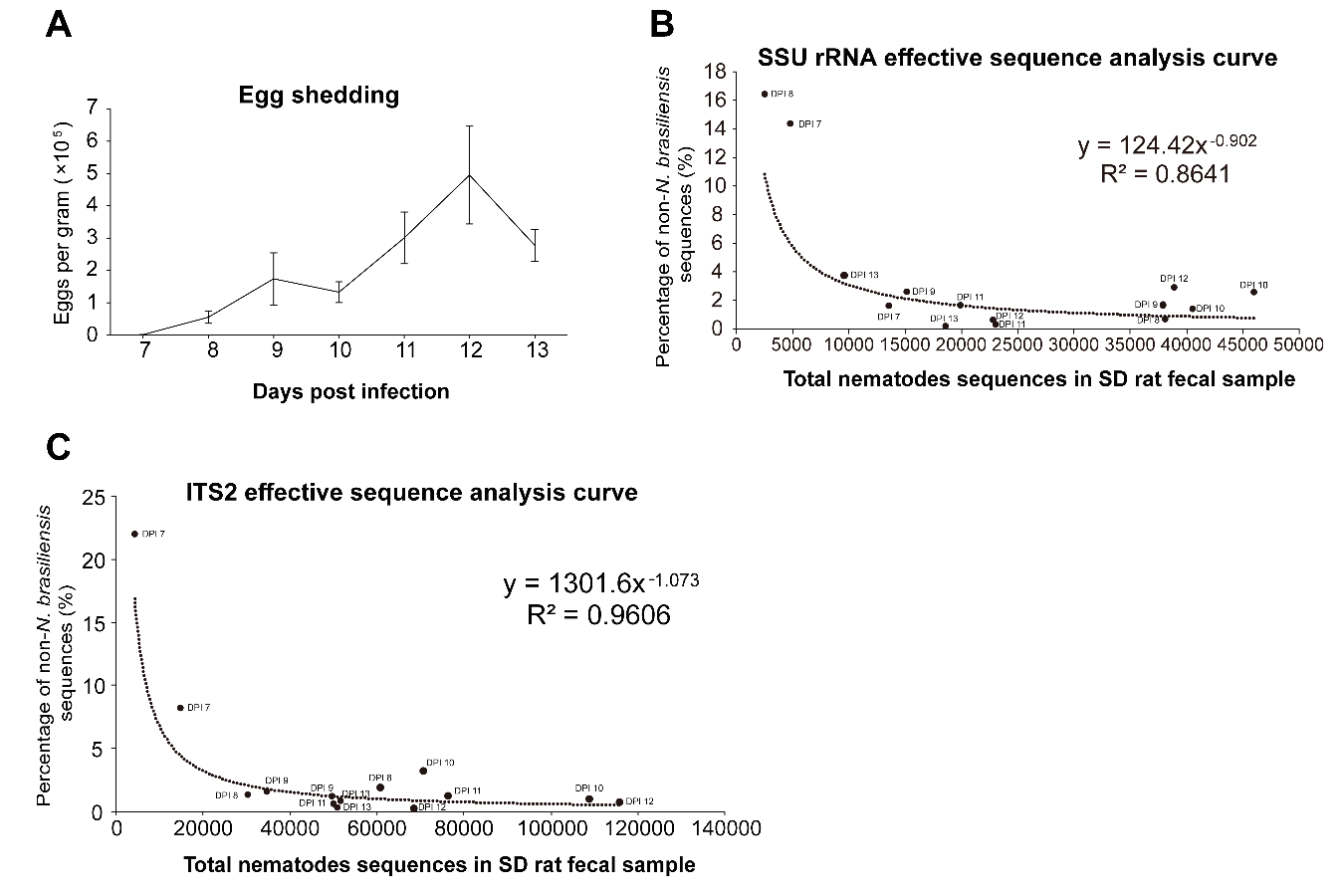


**Figure S1 Standard curve of egg shedding, SSU rRNA and ITS2 effective sequence analyses.** (A) Analysis of egg shedding of *N. brasiliensis* from SD rats. (B) SSU rRNA effective sequence analysis curve of *N. brasiliensis* from SD rats. (C) ITS2 effective sequence analysis curve of *N. brasiliensis* from SD rats.

**Table S1 Description of 512 fecal samples collected from 121 species of captive wild animals at three sampling sites in southern China**

| Location  (No. samples) | Animal type | | Animal species (scientific name, No. samples) | No. of specimens |
| --- | --- | --- | --- | --- |
| Zoo A  (272) | | Herbivore | Dama (*Dama dama*, 13), Kangaroo (*Macropus* sp., 11), Impala (*Aepyceros melampus*, 11), Big-eared goat (*Capra hircus*, 8), Debao pony (*Equus caballus*, 5), Giraffe (*Giraffa camelopardalis*, 5), Sambar deer (*Rusa unicolor*, 4), Asian elephant (*Elephas maximus*, 4), Javan deer (*Rusa timorensis*, 4), Argali (*Ovis ammon*, 4), Alpaca (*Vicugna pacos*, 4), Blue sheep (*Pseudois nayaur*, 3), Goral (*Naemorhedus goral*, 3), Sika deer (*Cervus nippon*, 2), Bornean red muntjac (*Muntiacus muntjak*, 2), African elephants (*Loxodonta africana*, 2), Dutch pony (*Equus caballus*, 2), Hippopotamus (*Hippopotamus amphibius*, 1), Addax (*Addax nasomaculatus*, 1) | 89 |
|  |  | Carnivore | South China tiger (*Panthera tigris* Amoyensis, 12), Lesser panda (*Ailurus fulgens*, 9), Malayan sun bear (*Helarctos malayanus*, 7), Leopard (*Panthera pardus*, 5), North Chinese tiger (*Panthera tigris* altaica, 4), Bengal white tiger (*Panthera tigris* tigris, 1), Jaguar (*Panthera onca*, 1), White lion (*Panthera leo*, 4), Lion (*Panthera leo*, 3), Black bear (*Ursus thibetanus*, 3), Brown bear (*Ursus arctos*, 2), Raccoon (*Procyon lotor*, 1) | 52 |
|  |  | Omnivore | Fragrance pig (*Sus scrofa*, 10), Fennec fox (*Vulpes zerda*, 6), Porcupine (*Hystrix cristata*, 4), Hedgehog (*Atelerix albiventris*, 2), Mongoose (***Herpestes****javanicus*, 1), Mara (*Dolichotis patagonum*, 1), Nutria (*Myocastor coypus*, 1) | 25 |
|  | | Non-human primate | Macaque (*Macaca mulatta*, 8), Francois's langur (*Trachypithecus francoisi*, 7), Golden monkey (*Rhinopithecus roxellana*, 4), Chimpanzee (*Pan troglodytes*, 4), Tibetan macaque (*Macaca thibetana*, 2), White-cheeked gibbon (*Nomascus siki*, 2), Ring-tailed lemur (*Lemur catta*, 2), Patas monkey (*Erythrocebus patas*, 2), Debrazza's monkey (*Cercopithecus neglectus*, 2), Silvery gibbon (*Hylobates moloch*, 1), Black-capped capuchin (*Cebus apella*, 1), Assamese macaque (*Macaca assamensis*, 1), Squirrel monkey (*Saimiri sciureus*, 1), Guereza (*Colobus guereza*, 1) | 38 |
|  |  | Reptile | Green iguana (*Iguana iguana*, 10), Tortoise (*Geochelone sulcata*, 6), Bearded dragon (*Pogona vitticeps*, 1), Rhinoiguana (*Cyclura cornuta*, 1) | 18 |
|  |  | Avian | Blue pheasant (*Lophura swinhoii*, 18), Golden pheasant (*Chrysolophus pictus*, 6), Swan (*Cygnus* ***cygnus*** 6), Silver pheasant (*Lophura nycthemera*, 3), Peafowl (*Pavo cristatus*, 5), Emu (*Dromaius novaehollandiae*, 4), Green peafowl (*Pavo muticus*, 2), Sulawesi hornbill (*Penelopides exarhatus*, 2), Cassowary (*Casuarius casuarius*, 1), Great pied hornbill (*Buceros bicornis*, 1), Reeves's pheasant (*Syrmaticus reevesii*, 1), Ostrich (*Struthio camelus*, 1) | 50 |
| Zoo B  (119) | | Herbivore | Elephant (*Elephas maximus*, 11), Giraffe (*Giraffa camelopardalis*, 10), Zebra (*Equus burchellii*, 9), Kangaroo (*Macropus* sp., 5), Oryx (*Oryx gazella*, 4), Alpaca (Vicugna pacos, 3), Impala (*Aepyceros melampus*, 3), Rhinoceros (*Dicerorhinus sumatrensis*, 2), Capybara (*Hydrochoerus hydrochaeris*, 1) | 48 |
|  |  | Carnivore | Anteater (***Myrmecophaga*** *tridactyla*, 3), Seal (*Phoca largha*, 2), Leopard (*Panthera pardus*, 2), North Chinese tiger (*Panthera tigris* altaica, 2), Lesser panda (*Ailurus fulgens*, 1), Sea lion (*Neophoca cinerea*, 1), Black leopard (*Panthera pardus* fusca, 1), Leopard cat (*Prionailurus bengalensis*, 1), Panthera tigris (*Panthera tigris*, 1), South China tiger (*Panthera tigris* amoyensis, 1), Bengal white tiger (*Panthera tigris* tigris, 1) | 16 |
|  |  | Non-human primate | Ring-tailed lemur (*Lemur catta*, 12), Golden monkey (*Rhinopithecus roxellana*, 5), White-nosed bearded sakis (*Chiropotes albinasus*, 4), Gibbon (*Hylobates* sp., 4), Debrazza's monkey (*Cercopithecus neglectus*, 4), Pig-tailed macaque (*Macaca nemestrina*, 2), Baboon (*Papio hamadryas*, 2), Mandrill (*Mandrillus sphinx*, 2), Spider monkey (*Ateles paniscus*, 1), Chimpanzee (*Pan troglodytes*, 1), Orangutan (*Pongo abelii*, 1), Black-capped capuchins (*Cebus apella*, 1), Patas monkey (*Erythrocebus patas*, 1), Ape (*Hominidae* sp., 1), Black mangabey (*Lophocebus aterrimus*, 1), Squirrel monkey (*Saimiri sciureus*, 1) | 43 |
|  | | Avian | Penguins (*Pygoscelis* sp., 4), Red-breasted parakeet (*Psittacula alexandri*, 1), White cockatoo (*Cacatua alba*, 1), Hornbill (*Anthracoceros* sp., 1), Macaws (Psittacidae, 1), Malabar pied hornbill (*Anthracoceros coronatus*, 1), Toucans (*Ramphastos toco*, 1), Sulphur-crested cockatoo (*Cacatua galerita*, 1), Eclectus parrot (*Eclectus roratus*, 1) | 12 |
| WRC C  (121) | | Herbivore | Black goat (*Capra aegagrus*, 6), Bornean red muntjac (*Muntiacus muntjak*, 4) | 10 |
|  |  | Carnivore | Chinese pangolin (*Manis pentadactyla*, 6) | 6 |
|  |  | Non-human primate | Macaque (*Macaca mulatta*, 13), Stump-tailed monkey (*Macaca arctoides*, 4), Cynomolgus monkey (*Macaca fascicularis*, 2) | 19 |
|  |  | Reptile | African spurred tortoise (*Geochelone sulcata*, 3), Geochelone radiata (*Astrochelys radiata*, 2), Python (*Malayop****ython*** *reticulatus*, 2), Green lizard (*Lacerta viridis*, 1) | 8 |
|  | | Avian | Peafowl (*Pavo cristatus*, 20), Silver pheasant (*Lophura nycthemera*, 15), Crane (*Grus* sp., 10), African grey parrot (*Psittacus erithacus*, 6), Hill myna (*Gracula religiosa*, 6), Yellow-bellied tragopan (*Tragopan caboti*, 4), Pigeon (*Columba livia*, 4), Monk parakeet (*Myiopsitta monachus*, 3), Eclectus parrot (*Eclectus roratus*, 3), Macaws (*Ara* sp., 2), Red-necked amazon (*Amazona arausiaca*, 1), Pacific parrotlet (*Forpus coelestis*, 1), Budgerigar (*Melopsittacus undulatus*, 1), Lovebird (*Agapornis* sp., 1), Eagle (*Aquila* sp., 1) | 78 |
|  | |  | Total | 512 |

**Table S2 PCR primers for all loci**

| **Sites** | **Primers** | **Sequence (5´- 3´)** | **Fragment size (bp)** |
| --- | --- | --- | --- |
| ITS2 amplicon | NC1 | ACGTCTGGTTCAGGGTTGTT | ~350 |
|  | NC2 | TTAGTTTCTTTTCCTCCGCT |  |
| SSU rRNA amplicon | F | AGAGGTTCGAAGGCGATCAG | ~350 |
|  | R | GTCTCGYTCGTTATCGGAATWAAC |  |
| ITS1 of *Trichuris* spp. | ITS1_1417F | AGGGACCAGCGACACTTTC | ~1080 |
|  | ITS1_2505R | GAGTGTCACGTCGTTCTTCAAC |  |
|  | ITS1_1567F | GTTCTCGTGACTGGGAC | ~890 |
|  | ITS1_2462R | CTACGAGCCAAGTGATCC |  |
| SSU rRNA of *Ascaridia* and *Heterakis* species | F | AGTGCTTAACGCGGGCTTAT | ~724 |
|  | R | AAAGCACGCTGATTCCTCCA |  |

**Table S3 Six categories of representative sequences of SSU rRNA and ITS2**

| Categories | Representative sequences |
| --- | --- |
| SSU rRNA1 | ATACCGCCCTAGTTCTAACCGTAAACTATGCCTACTAGGTGTATGAATTATTAGTTATAATAATTTATGGACCTTCTCGGAAACGAAAGTCTTTCGGTTCCGGGGGAAGTATGGTTGCAAAGCTGAAACTTAAAGGAATTGACGGAAGGGCACCACCAGGAGTGGAGCCTGCGGCTTAATTTGACTCAACACGGGAAAACTCACCCGGGCCGGACACTATAAGGATTGACAGATTGATAGCTCTTTCATGATTTAGTGGTTGGTGGTGCATGGCCGTTCTTAGTTCGTGGATATGATTTGTCTG |
| SSU rRNA 2 | CAGACAAATCGCTCCACCAACTAAGAACGGCCATGCACCACTACCCACTGAATCAAGAAAGAGCTCTTGATCTGTCAATCCTCACAGTGTTCGGGACGGGTGAGTTTTCCCGTGTTGAGTCAAATTAAGCCGCATGCTCCACTCCTGGTGGTGCCCTTCCGTCAATTCCTTTAAGTTTCAGCTTTGCAACCATACTTCCCCCGGAACCGAAACACTTTGGTTTCCCGGAAGCTGCTCAGCGAGTCACAAAAAGAACGTCGCCGAATCGCTGGTTGGCATCGTTTACGGTCACAACTAGGGCGGTAT |
| SSU rRNA 3 | ATACCGCCCTAGTTCTGACCGTAAACGATACCAACTAGCGTTCCGCCGCTGGCAAGCACGCCTTGGCGGGCAGCTTCCCGGAAACGAAAGTTTTTCGGTTCCGGGGGAAGTATGGTTGCAAAGCTGAAACTTAAAGAAATTGACGGAAGGGCACCACCAGGAGTGGAGCCTGCGGCTTAATTTGACTCAACACGGGAAAACTCACCTGGCCCGGACACCGTGAGGATTGACAGATTGATAGCTCTTTCTTGATTCGGTGGTTGGTGGTGCATGGCCGTTCTTAGTTGGTGGAGTGATTTGTCTG |
| SSU rRNA 4 | CTGACAGACCAATCCACGAACCACGAACGGCCATGCGCCACCAACCACCGAATCAAGAAAGAGCTCTCAATCTGTCAATCCTCACGGTGTCCGGGCCAGGTGAGTTTTCCCGTGTTGAGTCAAATTAAGCCGCAGGCTCCACTCCTGGTGGTGCCCTTCCGTCAATCTCTTTAAGTTTCAGCTTTGCAACCATACTTCCCCCGGAACCGAAACACTTTCGTTTCCGGGAAGCTGCCCGCCAAGGCATATTTACCGATGACGGAACGCTAGTTGGTATCGTTTACGGTCAGAACTAGGGCGGTAT |
| SSU rRNA 5 | CAGACAAATCACTCCACCAACTAAGAACGACCATGCACCACCAATCACCAAATCATGAAAGAGCTATCAATCTGTCAATCCTTACGGTGTCTGGGCCAGGTGAGATTTCCCGTGTTGAGTCAAATTAAGCCGCAGGCTCCACTCCTGGTGGTGCCCTTCCGTCAATTTCTTTAAGTTTCAGCTTTGCAACCATACTCCCCCCGGAACCGAAAAACTTTCGTTTCCGGGAAGCTGCCCGCCTAAGCAAAAACGCCAAAGGCGGAACGCTAGTTGGTATCGTTTACGGTCAGAACTAGGGCGGTAT |
| SSU rRNA 6 | ATACCGCCCTAGTTCTGACCGTAAACGATACCAACTAGCGTTCCGTCGGCGGTAAATACGCCTTGACGGGCAGCTTCCCGGAAACGAAAGTCTTTCGGTTCCGGGGGAAGTATGGTTGCAAAGCTGAAACTTAAAGAAATTGACGGAAGGGCACCACCAGGAGTGGAGCCTGCGGCTTAATTTGACTCAACACGGGAAAACTCACCTGGCCCGGACACCGTGAGGATTGACAGATTGAGAGCTCTTTCTTGATTCGGTGGTTGGTGGTGCATGGCCGTTCTTAGTTGGTGGAGTGATTTGTCTG |
| ITS2 1 | AAATGATATGCTTAAGTTCAGCGGGTAATCACGCCTGAGCTCAGGTTGCATTATACAAATGATAAAAGAACATCGTCGCCATACAAGTCACTATCTAAGTCAATCTCAATATTCGCTGAGTACACTCAAATAGTGGCAACATGTTCATATCATTCAGGAATGTTACAATTTCATAACATCACGTTGCATGTATATGTTCTTGAACTGAAATGGGAATTGTCTAAAAGACAAATGCCATTTGACAAACAATGTTGAAATTAGCCACATTGTAGTATATGGTT |
| ITS2 2 | AAATGATATGCTTAAGTTCAGCGGGTAATCACGCCTGAGCTCAGGTTGCAATACAAATGATAACGAATACTACTATCTCCAACATGTCCCTGTTTAAATCAATCTCATATTCATTGAGTACATTTAAACAGTGATAATAGATTCATATCATTCAGAAATGTTCACATTACTATATGAACATTATGTTGCATATGTTATTCTTGAACTATAACGGGATTTGTCAAAACAAGTGTAGACAAATGCCATTAGACAAACAGTGTTAAAACGTTAGCCACACTGTAGTATATCGTT |
| ITS2 3 | AAGTGATATGCTTAAGTTCAGCGGGTAATCACGACTGAGCTCAGGTTGCATTGCAAATGACGTTTAACCGCAGTTGTCATACAGGCCCTGCCAAAGCATTCTTAGTCGCTAAACGCTTTGACAGTGACAACGAGGTCACGACATCCGTGCACCTTGGATTGCACCAAGATACCTGTTGCAATATATTTCTCATCTAGAACGAGGATCACATCAAATGCAAGCATCGTTCGACAAACAGTGTCACAAGCCACACTGTAGTATTTATA |
| ITS2 4 | AATGAATTTCTACAGTGTGGCTAACTCTAACACTGTTTGTCGAATGGTCATTGTCAAATATTGTGATGATTCCCATTTCAGTTCAAGAATAATACATGCAACATGATGTTAATGTTGTAATGACATTAATGTTCCTGTATGATGTGAACGTGTTGTTACTGTTTGAATGTACTCAGTGAATTTGAGATTGATTTAAACAGGGACATGTATAACAATAATGTTCAATTATCATTTGTATTGCAACCTGAACTCAGGCGTGATTACCCGCTGAACTTAAGCATATCACTT |
| ITS2 5 | AAATGATATGCTTAAGTTCAGCGGGTAGTCACGCCTGAGCTCAGGTTGCATTGCAAATAACAGAAACGTCGTTGTCATACTAGCCACTGCCGAAACGTTCTAAAGTCGGTAAACGATTCAGCAGCAACAACGAGTTTGCTGTCATTCAGCGTACGTTAGCAAACTAGCCAGCTAACGTACATGTTGCAATATATTCTGATCTAGAACGGGAATTGCTATAAGCAAGTGCCGTTCGACAAACAGTGTCACAAGCTACACTGTAGTAGATATA |
| ITS2 6 | AACGATAGAATACAGCATGGATTGTTTTGCTGTGTGTCGAGTGGTACTTGCTGTGTTACAGCGAATCCCATTCAAGTGAAGAAAGTTTGCAACATGGCTCTGTATTAGTGTCAAAGCTCCTGAATGATGTGAACGCGATTGTTGCCGTGTCGAATCGTACTCGATGAGAATGAGATGGATTTGATCGGGGACCTGCTGTGAACAATCGACTGCTCCGACATCATTTGCATTGCAACCTGAGCTCAGGCGTGACTACCCGCTGAACTTAAGCATATCACTT |

**Table S4 Evaluation of the classification effect of the amplicon**

| Amplicon | Feature sequence | Taxonomy | Animal species | Microscopic examination result |
| --- | --- | --- | --- | --- |
| SSU rRNA amplicon | S1 | *Strongyloides* spp. | Herbivore  Non-human primate,  *Manis pentadactyla* | *Strongyloides* spp. |
|  | S2 | *Trichuris* spp. | Non-human primate,  *Giraffa camelopardalis* | *Trichuris* spp. |
|  | S3 | Oxyuridomorpha | Reptile | Oxyurid |
|  | S4 | *Ascaridia* spp. / *Heterakis* spp. | Avian | *Ascaridia* spp. / *Heterakis* spp. |
|  | S5 | *Wellcomia* | *Hystrix* sp. | - |
|  | S6 | - | *Panthera tigris Amoyensis* | *Toxascaris leonina* |
| ITS2 amplicon | I1 | *Haemonchus contortus* | Herbivore | Strongylida |
|  | I2 | *Cooperia* spp. | Herbivore | Strongylida |
|  | I3 | *Oesophagostomum aculeatum* | *Macaca mulatta*,  *Macaca arctoides* | Strongylida |
|  | I4 | *Trichostrongylus colubriformis* | *Oryx gazella*  *Capra hircus* | Strongylida |
|  | I5 | *Ancylostoma ceylanicum* | *Panthera tigris Amoyensis Panthera tigris tigris,*  *Helarctos malayanus* | Strongylida |
|  | I6 | *Nematodirus helvetianus* | *Aepyceros melampus* | *Nematodirus* sp. |

Note: Classification was performed using blast results with sequence coverage of 100.0% and similarity of 99.0% or greater. – indicates several nematode species.
